# A nonhuman primate model of inherited retinal disease

**DOI:** 10.1172/JCI123980

**Published:** 2019-01-22

**Authors:** Ala Moshiri, Rui Chen, Soohyun Kim, R. Alan Harris, Yumei Li, Muthuswamy Raveendran, Sarah Davis, Qingnan Liang, Ori Pomerantz, Jun Wang, Laura Garzel, Ashley Cameron, Glenn Yiu, J. Timothy Stout, Yijun Huang, Christopher J. Murphy, Jeffrey Roberts, Kota N. Gopalakrishna, Kimberly Boyd, Nikolai O. Artemyev, Jeffrey Rogers, Sara M. Thomasy

**Affiliations:** 1 Department of Ophthalmology & Vision Science, School of Medicine, UC Davis, Sacramento, California, USA.; 2 Human Genome Sequencing Center and Department of Molecular and Human Genetics, and; 3 Department of Biochemistry and Molecular Biology, Baylor College of Medicine, Houston, Texas, USA.; 4 Department of Surgical and Radiological Sciences, School of Veterinary Medicine, University of California-Davis, Davis, California, USA.; 5 California National Primate Research Center, Davis, California, USA.; 6 Department of Ophthalmology, Cullen Eye Institute, Baylor College of Medicine, Houston, Texas, USA.; 7 EyeKor Inc., Madison, Wisconsin, USA.; 8 Department of Molecular Physiology and Biophysics, and; 9 Department of Ophthalmology and Visual Sciences, The University of Iowa Carver College of Medicine, Iowa City, Iowa, USA.

**Keywords:** Ophthalmology, Genetic diseases

## Abstract

Inherited retinal degenerations are a common cause of untreatable blindness worldwide, with retinitis pigmentosa and cone dystrophy affecting approximately 1 in 3500 and 1 in 10,000 individuals, respectively. A major limitation to the development of effective therapies is the lack of availability of animal models that fully replicate the human condition. Particularly for cone disorders, rodent, canine, and feline models with no true macula have substantive limitations. By contrast, the cone-rich macula of a nonhuman primate (NHP) closely mirrors that of the human retina. Consequently, well-defined NHP models of heritable retinal diseases, particularly cone disorders that are predictive of human conditions, are necessary to more efficiently advance new therapies for patients. We have identified 4 related NHPs at the California National Primate Research Center with visual impairment and findings from clinical ophthalmic examination, advanced retinal imaging, and electrophysiology consistent with achromatopsia. Genetic sequencing confirmed a homozygous R565Q missense mutation in the catalytic domain of PDE6C, a cone-specific phototransduction enzyme associated with achromatopsia in humans. Biochemical studies demonstrate that the mutant mRNA is translated into a stable protein that displays normal cellular localization but is unable to hydrolyze cyclic GMP (cGMP). This NHP model of a cone disorder will not only serve as a therapeutic testing ground for achromatopsia gene replacement, but also for optimization of gene editing in the macula and of cone cell replacement in general.

## Introduction

High-acuity vision is dependent on the cone photoreceptor visual pathway in all species with macular function. In humans with retinal disease, the final common pathway of visual acuity loss is cone photoreceptor dysfunction, or the pathway carrying cone-mediated information. Most cases of retinal blindness in the population are age related, secondary to systemic metabolic disorders, or due to retinal vascular disease. Since damage to cone photoreceptors underlies the mechanism of visual acuity loss in each of these processes, understanding the biology of this retinal cell type is paramount to promoting retinal health and preserving vision. One of the most valuable sources of information regarding cone photoreceptor biology is the disease circumstance in which cones malfunction.

Cone disorders can be classified into achromatopsia, cone dystrophy, cone-rod dystrophy, color vision impairment, and macular dystrophies such as Stargardt disease. Phototransduction is the most commonly affected cellular process in cone disorders. This process depends on at least 10 genes that code for proteins in photoreceptor outer segments. Other cellular processes that can go awry in cone disorders include photoreceptor development, membrane morphogenesis and maintenance, protein trafficking to the outer segment, cellular signal transduction, or the recycling of photopigment in the visual cycle. Achromatopsia is caused by mutations in cone phototransduction machinery. Greater than 90% of achromatopsia cases can be explained by causative mutations in the 5 genes implicated in this disease (1). However, cone-rod dystrophies, though associated with 17 different genes (2), can be attributed to a specific causative mutation in only 21% of clinical cases. Similarly, 8 cone dystrophy genes have been identified, but these explain only 25% of clinical diagnoses (3).

At least 42 genes have been identified that disrupt cone function, and mouse models are available for the majority of these (3). About half of the mouse models have had therapeutic strategies evaluated. Accordingly, some progress has been made in understanding the pathophysiology of disease in a variety of cone disorders. However, no disease models exist in the context of high-acuity vision. The central human retina has a high cone density, which the mouse lacks, while outside of the central 10 degrees, the rod-to-cone ratio is similar in both species (4). Furthermore, mice and other nonprimate mammals such as cats and dogs all lack macular architecture. The macula is a cone-rich specialized retinal structure that is the source of all high-acuity visual functioning. Macular structure and function are most similar between humans and nonhuman primates (NHPs). Thus, NHP models of macular disease are highly desirable. Examples of individual NHPs with presumed inherited retinal disease have been described, but no genetic cause has been reported (5, 6). We are not aware of any previous studies of NHP retinal degeneration in which the causative mutation has been identified. There have been very few validated NHP models of Mendelian genetic disorders reported in the literature (7–10). Herein, we report severe vision impairment secondary to a homozygous missense mutation (R565Q) in the catalytic domain of the cone-specific phosphodiesterase 6α enzyme, encoded by the PDE6C gene in rhesus macaques from the California National Primate Research Center (CNPRC) at UC Davis. Originally identified by behavioral tests and observations, we report their adaptive behaviors, ocular examination findings, results of advanced ophthalmic imaging, and retinal electrophysiology. Furthermore, we report the genetic screening, identification of the causative mutation, predicted structural abnormality in the enzyme, stability of the protein product in vitro, and degree of biochemical function. These animals, genealogically related and presumably the descendants of a common founder, have characteristic signs, symptoms, and functional testing results that parallel humans with PDE6C-related cone photoreceptor disease.

## Results

### Visually impaired rhesus macaques show signs of achromatopsia.

The Population and Behavioral Health Services (PBHS) staff at CNPRC observed behaviors attributed to visual compromise in 4 rhesus macaques (3 females and 1 male, ages 2–11 years). These primates had similar signs of visual compromise, including decreased ability to navigate new environments and subsequently using their forelimbs to feel for walls and obstacles. When the primates were housed in smaller enclosures that were familiar to them, their visual impairment was less obvious. These macaques appear to elicit tolerant responses from their group members to behaviors that would normally trigger antagonism (e.g., direct gaze at close proximity). When housed in groups in large enclosures, some of these subjects were observed to be led by unaffected members holding their hand. Behavioral abnormalities such as eye poking and saluting (covering a portion of the eye with the hand to decrease the visual stimulus) were also observed. The aforementioned behaviors were particularly evident when the animals were in normal illumination settings such as outdoor daylight or indoor fluorescent lighting. These observations prompted the PBHS staff to perform additional behavioral testing and to request that the veterinary staff examine these primates for ocular disease. Thus, 2 affected rhesus macaques and 2 age-matched control animals were placed in unfamiliar indoor enclosures under bright light conditions. The control animals were able to easily identify perches above them and food items such as grapes on the floor. By contrast, visually impaired subjects in the unfamiliar enclosure were found to avoid the floor, cover their eyes under normal indoor illumination conditions, and use their hands to confirm the presence of physical objects around them (Supplemental Video 1; supplemental material available online with this article; https://doi.org/10.1172/JCI123980DS1).

**Figure 1:**
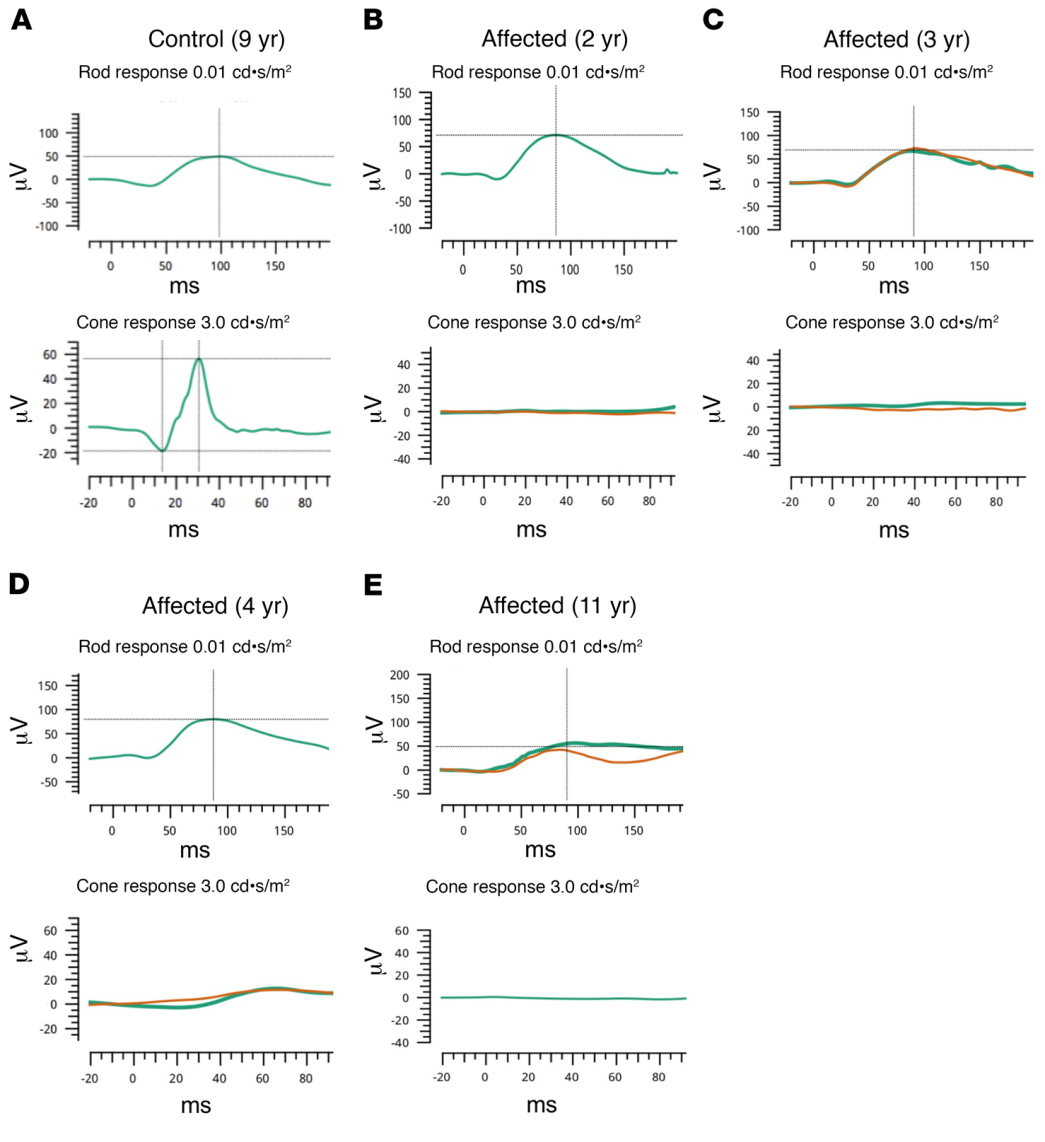
Visually impaired rhesus macaques have electroretinography tracings characteristic of achromatopsia. An example of an electroretinogram (ERG) tracing from the eye of an unaffected macaque (**A**) is shown. Normal amplitude and latency is observed in the control case in both the rod-mediated (**A**, top row; dark adapted state, 0.01 cd•s/m^2^ stimulus) and cone-mediated pathways (**A**, bottom row; light adapted state, 3.0 cd•s/m^2^ stimulus). Affected subjects (**B**–**E**) noted to have visual impairment have relatively normal rod-mediated waveforms with perhaps subnormal amplitudes (**B**–**E**, top row). By contrast, affected animals (**B**–**E**, bottom row) have nonmeasurable cone-mediated recordings at 2 (**B**), 3 (**C**), 4 (**D**), and 11 (**E**) years of age, respectively. Green traces indicate initial testing. Orange lines indicate repeated testing during the same recording session. μV: microvolts, ms: milliseconds.

**Figure 2:**
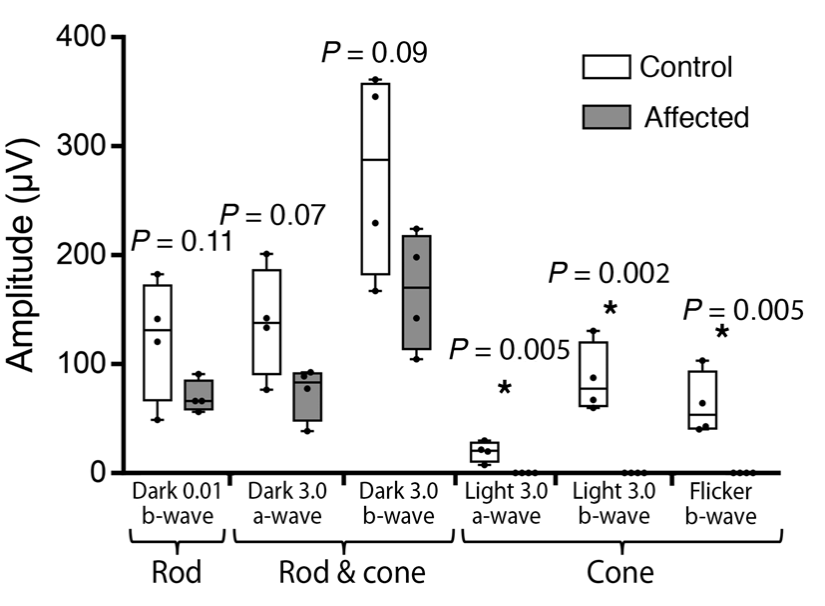
Quantitative comparison of electroretinography shows absent cone-mediated and subnormal rod-mediated amplitudes. The absolute value of the ERG amplitudes (μV: microvolts) of both a- and b-waves from control and affected subjects was quantified and is graphically depicted. Under dark adapted conditions, using a 0.01 cd•s/m^2^ stimulus, the rod system was tested and found to be subnormal in affected subjects when compared with unaffected controls. The rod and cone combined ERG, measured under dark adapted conditions using a 3.0 cd•s/m^2^ stimulus, showed moderately reduced responses in affected subjects compared with controls. The cone-mediated pathway was tested in the light-adapted state using a single flash 3.0 cd•s/m^2^ stimulus, and also a flicker (30 Hz) with the same stimulus and showed virtually undetectable responses in the affected subjects (*n* = 4 in each group, *P < 0.05, Student’s *t* test). Whiskers represent minimum and maximum. Boxes represent interquartile range. Line represents the median, and dots represent data points.

Four affected macaques and 4 control subjects were sedated and received eye examinations involving clinical tests and imaging similar to those performed on human patients in the UC Davis Eye Center. Eye examinations in all subjects revealed symmetric pupillary light reflexes and unremarkable anterior segments. The subjects were then dilated and dark adapted for electrophysiologic testing. Scotopic and photopic full-field electroretinograms (ERGs) were performed on controls (Figure 1A) and demonstrated normal rod and cone responses. The recordings from the affected subjects showed a relatively normal rod response, but a completely absent cone response (Figure 1, B–E). When combining the responses of the visually impaired animals, a modest reduction in rod-mediated amplitudes discerned in these measurements did not reach statistical significance (Figure 2, left). Predictably, the combined maximal response of the ERG under dark adapted conditions, a function of both rod- and cone-mediated pathways, was reduced owing to the absence of a cone-mediated contribution (Figure 2, center). The cone-specific amplitudes measured under light adaptation showed no recordable signal in affected subjects in the single-flash ERG and in the photopic flicker when compared with controls (Figure 2, right).

### Rhesus macaques show clinical signs consistent with achromatopsia.

**Figure 3:**
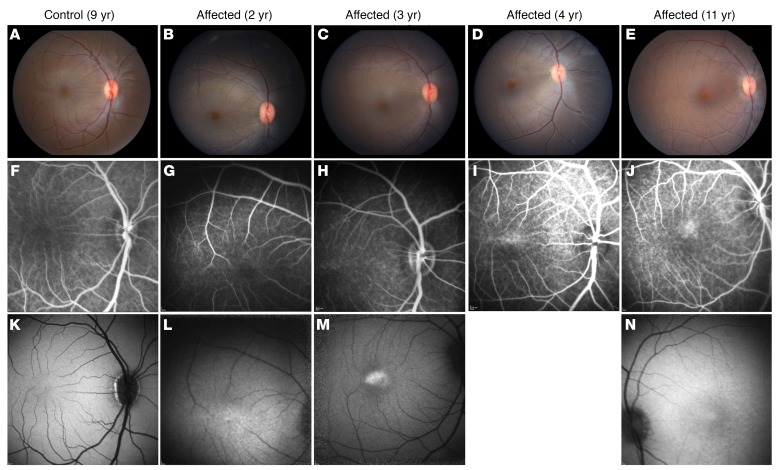
Noninvasive retinal imaging of affected visually impaired rhesus macaques shows evidence of slowly progressive macular atrophy consistent with achromatopsia. Color fundus photography (**A**–**E**), fluorescein angiography (**F**–**J**), and fundus autofluorescence (**K**–**N**) were obtained in unaffected control and affected visually impaired subjects. An example of a control subject is shown (**A**, **F**, **K**; age 9 years), demonstrating normal posterior pole findings. The fundus photos of affected animals show a largely normal macular appearance, but with prominent foveal pigmentation (**B**–**E**). The fluorescein angiogram in affected subjects demonstrated normal retinal vasculature at ages 2 (**G**) and 3 years (**H**), but the appearance of a bullseye pattern of foveal staining surrounded by parafoveal hypofluorescence was evident by age 4 years (**I**), and even more obvious by age 11 years (**J**). Fundus autofluorescence shows normal macular autofluorescence at age 2 years (**L**). At age 3 years (**M**) there is prominent foveal hyperautofluorescence, and development of an annulus of hypoautofluorescence centered on the fovea by age 11 years (**N**) which corresponds to the bullseye pattern seen on fluorescein angiography. Imaging software failure precluded fundus autofluorescence in the 4-year-old subject. Color fundus images were taken with a 50 degree lens. Fluorescein angiography and fundus autofluorescence images were taken with a Heidelberg Spectralis device using the default image size of 30 degrees.

Indirect ophthalmoscopy in affected animals revealed subtle posterior segment changes in affected subjects. In particular, the foveal center appeared more pigmented (Figure 3, B–E) when compared with control subjects (Figure 3A). This finding may be due to thinning of the fovea, which may lead to increased retinal translucency, and a more pronounced appearance of the underlying retinal pigmented epithelium (RPE).

**Figure 4:**
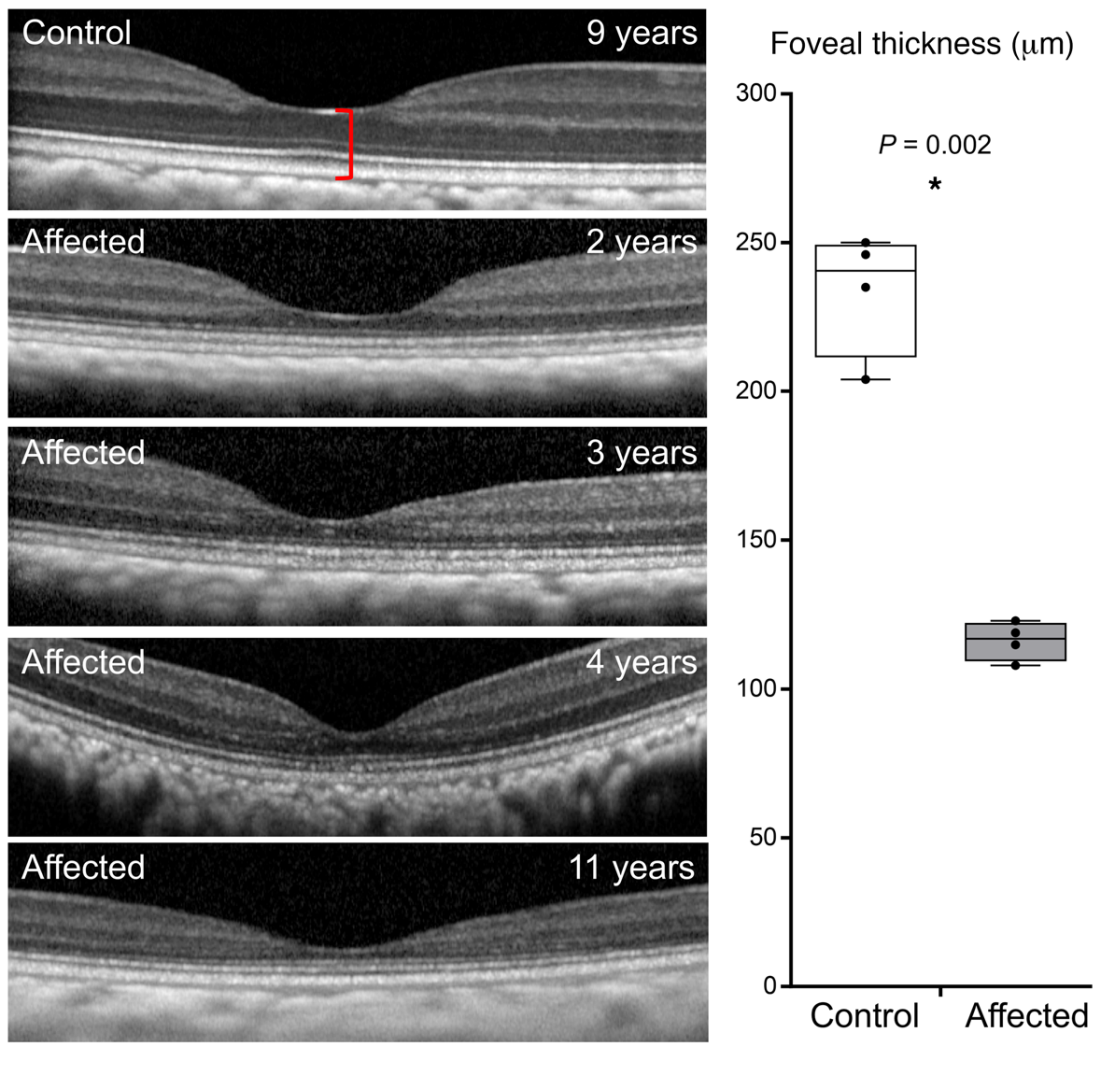
Foveal thinning is observed in visually impaired affected rhesus macaques. Spectral domain optical coherence tomography (SD-OCT) of the foveal center is shown from a control animal and from the 4 affected animals with the PDE6C^R565Q^ mutation. Quantification of the thickness of the foveal center (box plot, right), measured from the internal limiting membrane to Bruch’s membrane (red caliper in control panel). There is a trend toward foveal thinning with age among the 4 affected animals (123 μm at age 2 years, 119 μm at age 3 years, 115 μm at age 4 years, 108 μm at age 11 years) (*n* = 4 in each group, *P < 0.05). Whiskers represent minimum and maximum. Boxes represent interquartile range. Line represents the median, and dots represent data points.

**Figure 5:**
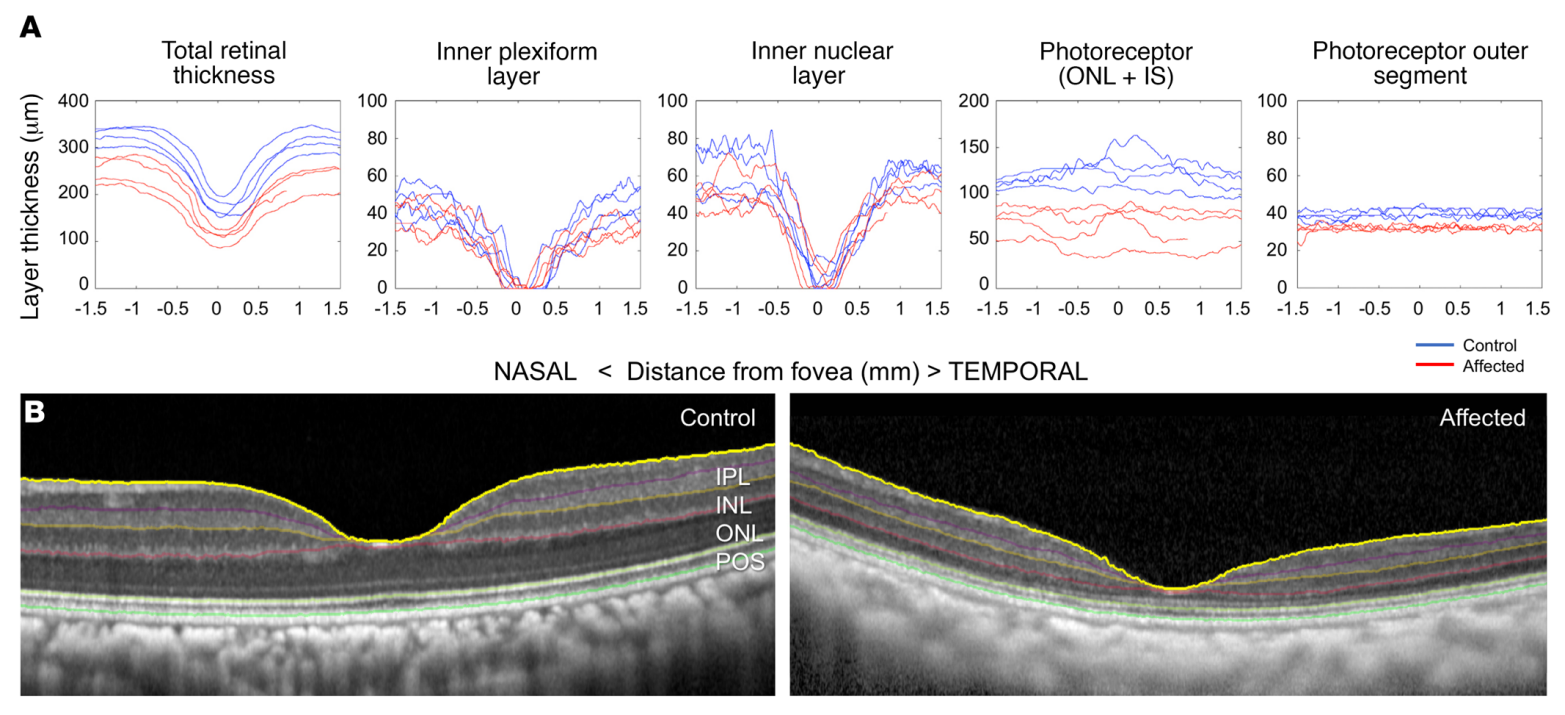
Retinal thinning in affected visually impaired animals is due to cone photoreceptor loss. Comparing the topographical thickness of retinal layers in the parafoveal region (**A**, control blue, affected red) shows thinning of the retina. The reduced thickness is secondary to thinning in the outer nuclear layer and photoreceptor outer segments. The other retinal layers are of similar thickness to unaffected controls. The thickness of retinal layers was measured by semiautomated segmentation of SD-OCT horizontal scans through the foveal center (**B**, unaffected control left, affected right). Inner plexiform layer (IPL), inner nuclear layer (INL), outer nuclear layer (ONL + inner segments), photoreceptor outer segments (POS). *n* = 4 for each group.

Furthermore, a subtle but characteristic bullseye maculopathy was identified in the affected macaques (Figure 3, G–J) using fluorescein angiography (FA). This was not present in control subjects (Figure 3F). The macular abnormalities were also seen using blue fundus autofluorescence (FAF) (Figure 3, L–N) in affected macaques, whereas control subjects had normal FAF (Figure 3K). The abnormal foveal appearance on color fundus photographs, FA, and FAF were corroborated using spectral-domain optical coherence tomography (SD-OCT). The foveal center-point thickness (red caliper in Figure 4A), was reduced in affected subjects when quantitatively compared with controls (Figure 4B). This measurement confirmed the clinical suspicion of foveal thinning in affected subjects based on the pronounced appearance of the foveal RPE. In order to determine which layers of the retina were affected, semiautomated segmentation of the parafoveal region was performed on horizontal foveal b-scans (Figure 5B). Comparison of affected subjects with controls revealed that the global retinal thinning results from thinning of the outer nuclear layer (ONL) and photoreceptor outer segments (Figure 5A). There were no statistically significant differences in the other retinal layers (Table 1). In aggregate, these findings support the clinical diagnosis of achromatopsia in the visually impaired rhesus macaques at CNPRC.

### Affected macaques are homozygous for a missense mutation in PDE6C.

**Figure 6:**
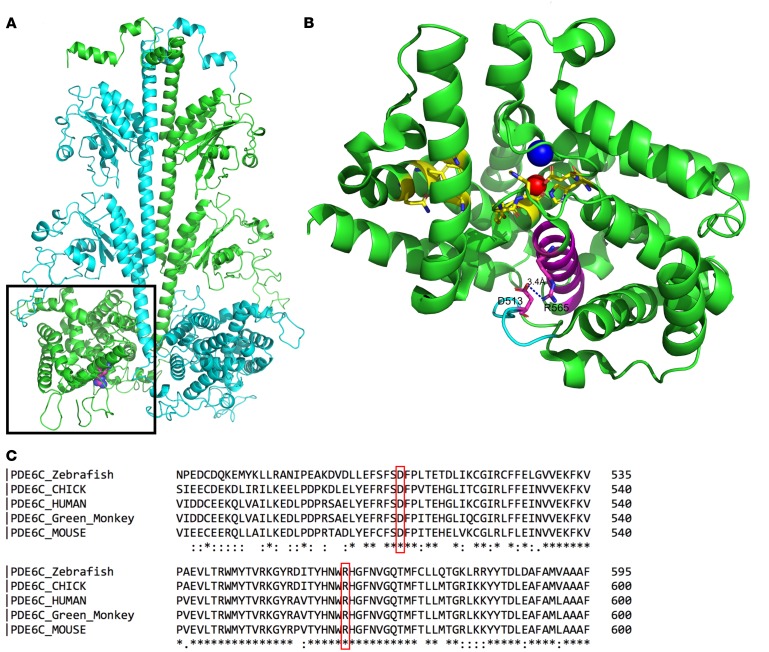
The R565Q mutation in PDE6C alters a conserved arginine in the catalytic domain. (**A**) A model of human PDE6C protein dimer was generated using SWISS-MODEL based on the study by Zeng-Elmore et al. (11). The boxed portion is a catalytic domain and the R565 residue is highlighted (depicted in sphere, carbon in magenta). (**B**) Magnified view around residue R565 and its putative interaction with D513. R565 and D513 are shown as sticks, colored by element (carbon in magenta). Loop 509–516 is shown in cyan and helix 565–579 is shown in magenta. Catalysis-related residues are shown as sticks, colored by element (carbon in yellow). Zinc and magnesium ions are shown as red and blue spheres, respectively. Original data are from the Protein Data Bank (accession ID: 3jwr). (**C**) Protein sequence alignment shows that both D513 and R565 are highly conserved (boxed).

DNA was extracted from blood samples from the 4 affected subjects and 2 of these subjects were sequenced using next generation sequencing technology. Based on the clinical diagnosis, attention was focused on a list of 43 candidate nonsyndromic cone disorder genes (3). Sequencing results and confirmatory genotyping revealed that all 4 affected subjects were homozygous for the same missense genetic variant in one of the candidate genes, PDE6C, which codes for the alpha-prime subunit of cone phosphodiesterase 6C. This protein hydrolyzes intracellular cyclic GMP (cGMP) and thus causes cGMP-gated channels to close and the photoreceptor plasma membrane to hyperpolarize, which is critical to the phototransduction cascade. The mutation changes an arginine to a glutamine at position 565 (R565Q). This alteration is within the catalytic domain of the enzyme, and specifically within the first metal binding motif ^562^HNWRH^566^, which is critical in all PDE families for hydrolysis of cyclic nucleotides. Moreover, the arginine at position 565 itself is highly conserved across vertebrates (Figure 6C), not only in PDE6 but also in other PDEs such as PDE5 (Supplemental Figure 2), suggesting it is playing an important functional role (Figure 6, A and B). A model of human cone PDE6C based on the model of the rod PDE6 catalytic dimer (11) indicates that R565 likely interacts with the aspartate at position 513 (Figure 6B). Likewise, D513 is highly conserved in the PDE6 family (Figure 6C). An R to Q substitution is predicted to be deleterious by multiple in silico prediction software tools, such as Combined Annotation Dependent Depletion (CADD), Sorting Intolerant From Tolerant (SIFT), and Polymorphism Phenotyping (PolyPhen). With the LiftOver tool of UCSC Genome Browser (12) and ANNOVAR (13), the corresponding human variant was identified in chr10:95400271 G>A (GRCh37), NM_006204, exon13, c.G1694A, p.R565Q, and annotated. The R565Q variant alters an amino acid site highly conserved in vertebrates with a phyloP100way_vertebrate_rankscore of 0.79983, and a phastCons100way_vertebrate_rankscore of 0.71417 (the scores are in the scale of 0 to 1, annotated by dbNSFPv2.9) (14, 15). The human mutation is considered damaging by 10 prediction algorithms, including SIFT_phred (D), Polyphen2_HDIV_pred (D), Polyphen2_HVAR_pred (D), LRT_pred (D), MutationTaster_pred (D), MetaSVM_pred (D), MetaLR_pred (D), VEST3_rankscore (0.84943), PROVEAN_pred (D), CADD_raw_rankscore (0.93161), annotated by dbNSFPv2.9 (Supplemental File 1). The corresponding human variant is only found in one individual in heterozygous status out of 123,101 individuals in the gnomAD database (16), which can serve as a control data set, suggesting the mutation is extremely rare in the human population.

**Figure 7:**
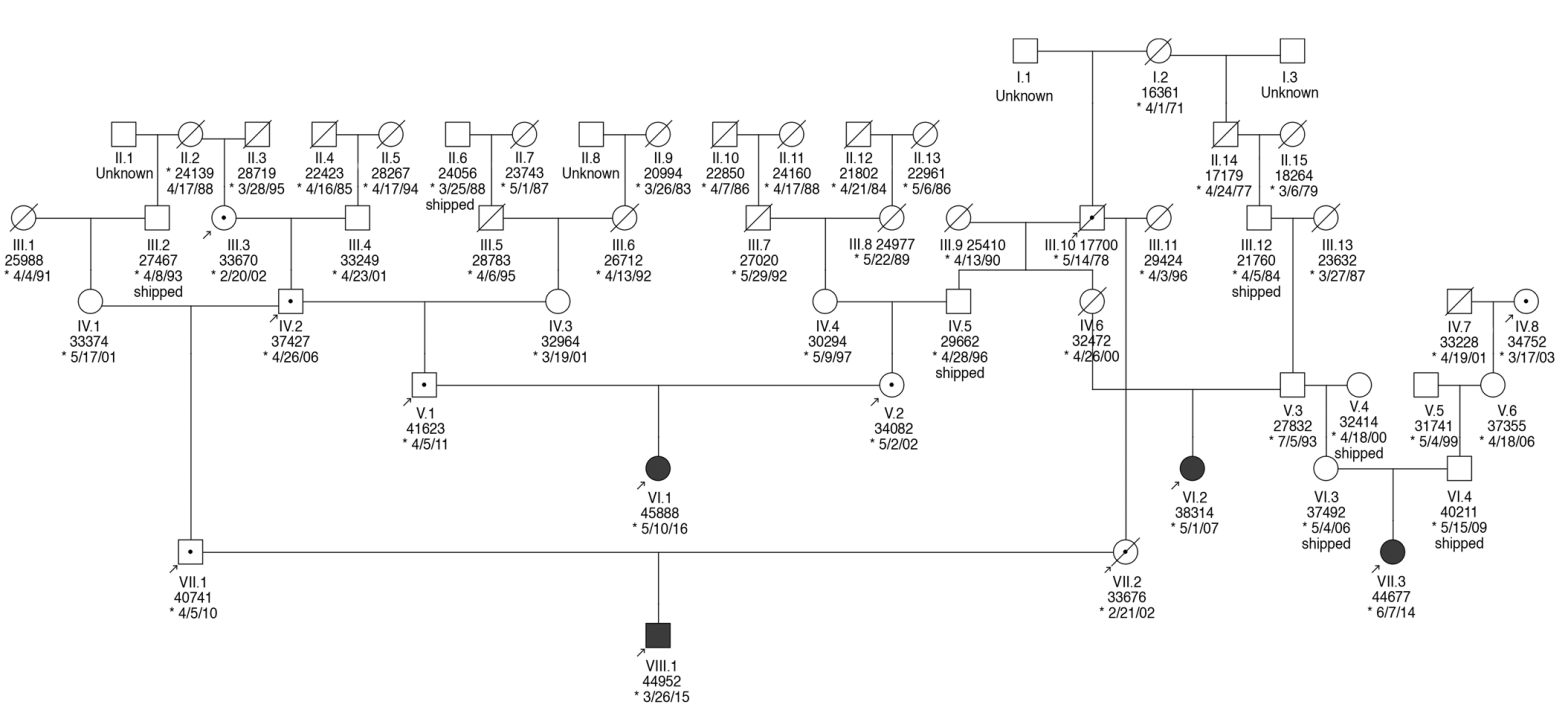
Pedigree of affected rhesus macaques. Partial pedigree of the 4 affected rhesus macaques (filled). Genotyping was conducted and all 4 affected subjects were determined to be homozygous for the R565Q mutation in PDE6C. In addition, 8 unaffected related individuals (arrows) were genotyped and are heterozygous carriers noted with a dot. Squares represent males, and circles represent females. Crossed line represents that the individual is no longer available within the colony. * Date of birth.

To further test the pathogenicity of this variant, segregation testing was performed. Consistent with the recessive inheritance model, genotyping of the parents of 2 affected individuals demonstrated that both parents were carriers (heterozygous for the mutated allele) and multiple unaffected siblings are either heterozygous for the mutation or WT (Figure 7). As part of a study to sequence a panel of 285 genes associated with human inherited retinal degeneration in 2000 NHPs, we screened genomes of related NHPs for the mutation. Genotyping of additional individuals has identified a total of 16 rhesus macaques at CNPRC that are heterozygous for the mutation in PDE6C. This variant is rare (*P* = 0.003) in our database of 527 randomly ascertained rhesus macaques from US research colonies. Mutations in PDE6C are responsible for approximately 1% of achromatopsia cases in humans (17) and 1% of cone dystrophy patients (18).

### The R565Q mutation in PDE6C impairs enzymatic function.

**Figure 8:**
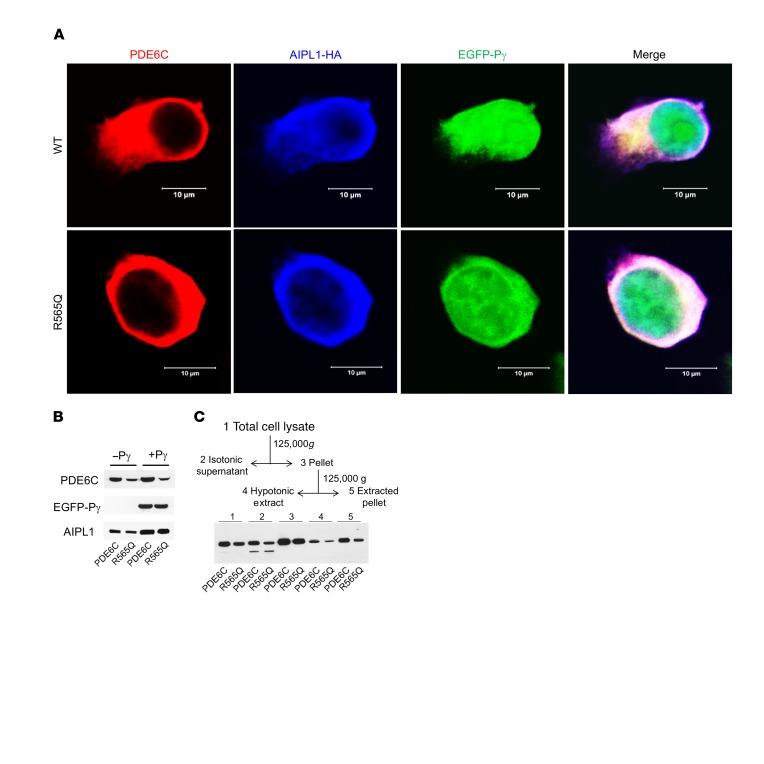
Coexpression of PDE6C and R565Q with AIPL1 and Pγ in HEK293T cells. (**A**) Confocal immunofluorescence images of HEK293T cells cotransfected with PDE6C or R565Q (red, anti-PDE6C) and the AIPL1-Pγ vector (AIPL1: blue, anti-HA; Pγ: green, EGFP fluorescence). (**B**) Immunoblot analysis of extracts of HEK293T cells cotransfected with PDE6C or R565Q and AIPL1 or the AIPL1-Pγ vector using anti-Flag (PDE6C), anti-EGFP (P), and anti-HA (AIPL1) antibodies. Lanes contain equal amounts of protein. From 4 similar experiments, the level of R565Q protein expression is reduced to 57% ± 9% (mean ± SEM) of that for PDE6C. (**C**) Scheme of fractionation of lysates from HEK293T cells cotransfected with PDE6C (or R565Q), AIPL1, and Pγ, and immunoblot analysis in the scheme fractions 1–5 with anti-Flag antibody. The findings suggest that the distributions of PDE6C and R565Q in HEK293T cells are qualitatively similar, but the amount of R565Q protein expression is reduced.

To test whether the R565Q allele affects PDE6C protein stability or function, in vitro assays were performed. Transfection of HEK293T cells with an expression plasmid containing cDNAs coding flag-tagged WT PDE6C or PDE6C^R565Q^ showed similar localization of the protein in the intracellular compartment (Figure 8A). The expression pattern of AIPL1-HA (the molecular chaperone of PDE6C) and EGFP-Pγ (a small regulatory subunit of PDE6C) was similar in cells transfected with WT PDE6C or PDE6C^R565Q^ (Figure 8A, Supplemental Figure 1). Immunoblots from lysates of these transfected cells showed PDE6C or PDE6C^R565Q^ protein in both samples, with or without the presence of Pγ, albeit the level of PDE6C^R565Q^ protein was reduced by about 2-fold compared with PDE6C (Figure 8B). These findings suggest that PDE6C^R565Q^ is translated into a relatively stable protein product that colocalizes with AIPL1 and Pγ in vitro in HEK293T cells. To assess the membrane distribution of R565Q in comparison to that of PDE6C, we used the fractionation scheme for HEK293T shown in Figure 8C. Association of PDE6 with the membrane requires proper C-terminal isoprenylation of its catalytic subunits (19). Dissociation of PDE6 from photoreceptor membranes can be achieved by extraction with hypotonic buffer (20). The immunoblot analysis indicates that PDE6C and R565Q are distributed in comparable proportions between soluble and membrane fractions following extractions with isotonic and hypotonic buffers.

**Figure 9:**
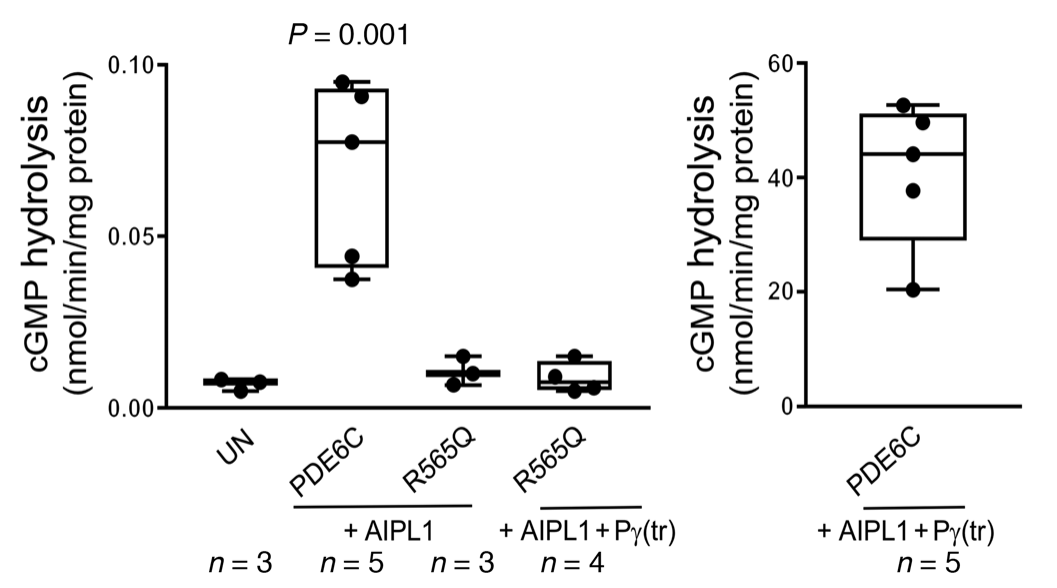
Catalytic activities of PDE6C and R565Q expressed in HEK293T cells. cGMP hydrolysis in extracts of HEK293T cells cotransfected with PDE6C or R565Q and AIPL1 alone or the AIPL1-Pγ vector (UN: untransfected control). Since Pγ is the inhibitory subunit of PDE6, the samples with coexpression of Pγ were treated with trypsin to selectively remove P (tr). The data suggest R565Q is catalytically inactive. The data were analyzed by 1-way ANOVA with Tukey’s multiple comparisons follow-up test. Whiskers represent minimum and maximum. Boxes represent interquartile range. Line represents the median, and dots represent data points. Exact *n* shown in figure.

In order to investigate the enzymatic consequences of the R565Q mutation, we used an assay to measure PDE6 activity in terms of hydrolysis of radiolabeled [^3^H]cGMP (nmol cGMP/min/mg protein). The activities were measured in HEK293T cell lysates following transfection with plasmids coding PDE6C in combinations with AIPL1 and Pγ. We determined that mutant PDE6C^R565Q^ had profoundly diminished enzymatic activity (indistinguishable from untransfected control cells) when compared with WT PDE6C, even in the presence of AIPL1 and Pγ (Figure 9). These findings support the conclusion that R565 is a critical amino acid in the catalytic domain required for proper enzymatic function of PDE6C.

## Discussion

Achromatopsia presents in humans in infancy with photophobia, nystagmus, hemeralopia, absent color discrimination, and poor visual acuity. The ERG findings in humans show absent cone amplitudes with preservation of the rod-mediated pathway, though we note that reductions of the latter have been reported (21, 22). While the clinical fundus examination is usually normal, evidence suggests achromatopsia is not a stationary disease. Variable degrees of subtle macular changes ranging from loss of the foveal reflex, macular pigmentation changes, and rarely atrophy of the RPE have been described (23). The findings on SD-OCT can be normal or exhibit variable degrees of disruption of the inner-segment/outer-segment junction (IS/OS junction) (i.e., the ellipsoid zone) (24–26). The foveal appearance in achromatopsia has been divided into 5 different SD-OCT patterns (27). The variable SD-OCT appearances do not correlate with genotype or age of the patients, suggesting that modifying genes or environmental factors may be responsible for the phenotypic differences. In achromatopsia, FAF ranges from normal to hyper- or hypoautofluorescence (28), and findings suggest that the macular changes can be progressive, with younger patients exhibiting hyperautofluorescence, and older patients showing hypoautofluorescence (29). This is consistent with the general concept that hyperautofluorescence is indicative of stressed RPE (30), and hypoautofluorescence correlates with nonfunctional or absent RPE (31).

We present a spontaneously occurring NHP model of achromatopsia presenting very early in life (1 year, 10 months) caused by R565Q mutation of the PDE6C gene, altering the catalytic domain of the PDE6C enzyme and nullifying its enzymatic hydrolysis of cGMP. Similar to human patients, PDE6C^R565Q^ macaques have unrecordable cone responses on ERG. It is possible that the rod responses in NHPs may begin to decline later in the disease course (Figure 1E, top panel), which also occurs in human achromatopsia patients (32, 33), as well as in 37% of cone dystrophy patients within 10 years of diagnosis (18). Hypotheses to explain the rod abnormalities have centered around fewer absolute numbers of rod photoreceptors, decreased length of their outer segments, and altered rod photoreceptor circuitry (34). The degree to which rod-photoreceptor dysfunction is present or develops in the affected NHPs reported here will be the subject of further study. As is typical in human cases, the clinical fundus appearance was relatively normal in PDE6C^R565Q^ macaques, and the macular SD-OCT had a normal IS/OS junction (ellipsoid zone). Consistent with prior reports of FAF in human achromatopsia, our younger subjects appeared to have hyperautofluorescent changes, while the oldest subject developed a bullseye appearance of hypoautofluorescence surrounding the foveal center. This may represent individual variability in the phenotype or may be indicative of disease progression. The tendency for FAF abnormalities to worsen with time in human patients, and the reduced rod amplitudes in our oldest subject, suggests that the latter explanation may be more likely. Our findings show that the PDE6C^R565Q^ macaques exhibit typical signs of achromatopsia, and have examination findings, ophthalmic imaging, and ERG recordings characteristic of this condition.

The PDE6C protein is chaperoned to the cone outer segment by AIPL1 and Pγ where it ultimately joins the other subunits of the enzyme on outer segment disks, which are infoldings of the cone outer segment membrane (20, 35, 36). Mutations in this gene are associated with achromatopsia in several human pedigrees, accounting for about 1% of clinical cases (17). A similar fraction of cone dystrophy and cone-rod dystrophy has been attributed to a mutation in this gene as well (18, 37, 38). The genotype-phenotype variability in patients with PDE6C mutations may be attributed to allelic differences, the effect of genetic modifiers, or environmental factors. In human subjects with PDE6C-associated achromatopsia, the fundus appears largely normal with subtle foveal pigmentary changes. Furthermore, the SD-OCT shows a thinned foveal center with localized hyporeflectivity indicative of subretinal fluid. In addition, the FAF is altered and the ERG shows absent cone responses from an early age (39, 40). The phenotype of our affected subjects is identical to the human patients with PDE6C-associated disease, with the exception of the foveal hyporeflective lesion seen on SD-OCT. In patients with PDE6C-associated achromatopsia, mutations can lead to protein degradation, cellular mislocalization, or enzymatic dysfunction (41). Consistent with human cases exhibiting mutations in the catalytic domain of this enzyme, the macaque PDE6C^R565Q^ protein is transcribed, translated, is relatively stable, but is incapable of hydrolyzing cGMP.

PDE6C^R565Q^ macaques are ideal for evaluation of therapeutic strategies for achromatopsia, and potentially for other related retinopathies. Small and large animal models of spontaneously occurring achromatopsia exist and have been used successfully for gene therapy (42). Many groups have reported the results of gene therapy strategies in animal models of achromatopsia. Gene replacement can improve the function of cone cells in achromatopsia mouse models with CNGB3- (43), CNGA3- (44–46), and GNAT2-associated disease (47). Treatment of dogs with achromatopsia secondary to CNGB3 mutation have shown durable rescue of cone function after viral-mediated gene replacement for at least 33 months (48). Sheep with mutation in CNGA3 have also been successfully treated with a similar approach (49). As a result of these successes in various animal models, phase I/II human clinical trials are in progress for CNGB3 (NCT02599922) and CNGA3 (NCT02610582) achromatopsia using AAV-mediated delivery of these genes (50). No gene replacement studies have been published concerning PDE6C in any species to date. PDE6C^R565Q^ NHPs could serve as an effective model not only to evaluate treatment of affected NHPs, but also to test and optimize viral-mediated treatment modalities by subretinal and intravitreal injection (51) of AAV2 carrying functional PDE6C. It may be worthwhile to perform preliminary studies in mice with spontaneous Pde6c mutations (17), or in Pde6c^R565Q^ engineered mice. In addition to gene replacement, PDE6C^R565Q^ NHPs may be a good model in which to test CRISPR/Cas9-mediated base editing or homology directed repair (CRISPR-HDR) to produce a functional genome-derived enzyme. Since no other genetically characterized, spontaneously occurring, inherited retinal degeneration models exist in NHPs, PDE6C^R565Q^ macaques may provide a good model to test CRISPR strategies once their efficiency and safety are elucidated.

Another possible application of this achromatopsia model takes advantage of the cone-silent state and relatively stable architecture of the macula to test cone replacement strategies. Cone replacement has suffered from challenges in the production of cone photoreceptors in vitro from retinal organoids, though some strategies have been reported to bias cells to adopt this fate (52–54). Cell transplantation to the retina is also undergoing a significant revitalization. The breakthroughs achieved in the last decade using mouse models (55) appear to be in part secondary to material transfer between transplanted cells and endogenous cells (56–58). Until recently, the eye was largely considered to be an immune privileged organ, but this notion is being challenged by recent studies showing that cell replacement strategies are improved in the immune suppressed state (59, 60). The PDE6C^R565Q^ macula may be an ideal testing ground for cone replacement, once the barriers of cone cell production and host immune suppression are overcome.

NHP models are particularly compelling due to their outstanding anatomic, developmental, physiologic, social, and genetic similarity to humans. This tremendously increases the relevance and translational potential of these models. Macaques share approximately 93% DNA sequence similarity with humans, and protein sequences are much more similar between humans and macaques than between humans and rodents (61). Furthermore, it has been demonstrated that rhesus macaques and humans share common susceptibility genes for age-related macular degeneration (AMD) and that cross-species genotype-phenotype correlations exist (62). The major challenge to using NHPs as models has been the lack of easy access to appropriate study subjects. NHPs, particularly Old World monkeys such as rhesus macaques, have been recognized as appropriate models for the study of ocular diseases (63). However, given the low prevalence of spontaneous disease, only a small number of individual animals are affected in even the largest research colonies, and their disease phenotype may go unrecognized. Recent reductions in the cost of DNA sequencing, and the resulting revelations concerning the amount of functional genetic variation segregating in rhesus macaque research colonies, now make broad surveys of these colonies for genetic mutations that display a disease phenotype practical (64).

We have identified a cone dystrophy in rhesus macaques at the CNPRC, characterized by marked photopic vision impairment, progressive macular thinning by optical coherence tomography, and absent cone function by electroretinography. These animals are homozygous for a missense mutation altering the catalytic domain of their PDE6C proteins, a gene associated with achromatopsia or cone dystrophy in humans. This NHP model of a cone disorder will be useful for evaluating gene replacement, gene editing, and cell-based replacement strategies. Continued characterization of the ophthalmic consequences and progression of disease in this IRD model, as well as expansion of this colony of macaques for the scientific community, will benefit various aspects of vision research. Identification of additional NHPs with spontaneously occurring IRDs constitutes an outstanding step forward that will generate new resources for further investigation. We anticipate that NHP models of retinal disorders will decrease the time required and improve the success rate for translation of gene- or cell-based therapies to human clinical trials.

## Methods

### Animals.

All the rhesus macaques (*Macaca mulatta*) studied were born and maintained at the California National Primate Research Center (CNPRC), which is accredited by the Association for Assessment and Accreditation of Laboratory Animal Care (AAALAC) International. All guidelines of the Association for Research in Vision and Ophthalmology Statement for the Use of Animals in Ophthalmic and Vision Research were followed. All studies were in accordance to the National Institutes of Health (NIH) *Guide for the Care and Use of Laboratory Animals* (National Academies Press, 2011). Ophthalmic examinations and phenotyping were performed according to an animal protocol approved by the Institutional Animal Care and Use Committee at UC Davis (IACUC protocol 20145). Standard procedures at the CNPRC involved routine physical examinations, which took place every 6 months under sedation. Blood samples were drawn routinely and preserved at –20°C for each animal. The Population and Behavior Health Services staff at the CNPRC observed outdoor animals for 20–30 minutes twice a month using all occurrence sampling (65). These observations were then used to identify social dynamics and abnormal behaviors among group members. Ophthalmic phenotyping of these animals was performed in response to the behavioral observations suggestive of reduced visual functioning.

### Ophthalmic phenotyping.

Animals were sedated with intramuscular injection of ketamine hydrochloride and dexmedetomidine. Ophthalmic examination included pupillary light reflex testing, external and slit lamp examination, measurement of intraocular pressure, and dilated funduscopy and streak retinoscopy.

### Ophthalmic imaging.

Color fundus photography was performed with a CF-1 Retinal Camera with a 50° wide-angle lens (Canon). SD-OCT with confocal scanning laser ophthalmoscopy was performed (Spectralis HRA+OCT). The Spectralis device was also used to obtain blue-peak fundus autofluorescence, as well as fluorescein angiography simultaneously with SD-OCT. The Heidelberg eye tracking Automatic Real-Time (ART) software was set at 25 scans for each b-scan. A horizontal high-resolution raster scan centered on the fovea was obtained. Semiautomated segmentation of retinal layers and foveal thickness measurements were performed on a horizontal line scan through the foveal center using a custom proprietary algorithm (EdgeSelect, EyeKor Inc.) and using the measurement tool in ImageJ (NIH), respectively. Animals were monitored by a trained technician and a CNPRC veterinarian at all times.

### Electroretinography.

While sedated, animals were dilated and dark adapted for 30 minutes. The RETevet instrument (LKC Technologies) coupled to an ERG-Jet electrode from the same vendor (item 95-011) was used to perform a standard flash ERG according to the approved protocol of the International Society for Clinical Electrophysiology of Vision (ISCEV) (66). This test consisted of electroretinography using flash stimuli at 0.01 cd•s/m^2^, 3.0 cd•s/m^2^, and 10.0 cd•s/m^2^, and oscillatory potentials (OPs), all in the dark-adapted state. In addition, flash stimuli of 3.0 cd•s/m^2^ intensity were used after 10 minutes in the light-adapted state, as well as a 30 Hz photopic flicker. Measurements were recorded and displayed using the manufacturer’s software.

### Genetic sequencing and analysis.

Whole-genome shotgun sequencing (WGS) was performed on 2 affected subjects following the protocol provided by the manufacturer. In brief, 1 μg genomic DNA was sheared using Covaris for 120 seconds and purified using Ampure XP beads. After end repair and A-tailing, indexed adaptors were added. The product was purified using Ampure XP beads and amplified using KAPA Hifi HotStart ready mix. After bead purification, the library was diluted and loaded onto the Illumina Novaseq6000 Sequencer for sequencing.

Sequencing reads from the 2 rhesus macaques were aligned to the rhesus Mmul_8.0.1 reference genome assembly using BWA mem (67) with an average mapped sequence depth of ×44.6. The GATK pipeline (68) was used to identify single nucleotide variants (SNVs) and insertions/deletions (indels). Variant effect predictor (VEP) (69) was used to annotate variants based on merged Ensembl and RefSeq gene models. Variants of interest in genes known to be involved in cone disorders (3) were identified for further analysis. The allele frequencies of the variants of interest were calculated for our reference database of whole-genome sequences from 537 rhesus macaques. The variants of interest were further examined by lifting the rhesus positions over to the orthologous human position and performing CADD analysis (70), which predicts the functional impact of variants. Direct PCR Sanger sequencing was conducted to further confirm the candidate mutation in PDE6C in each individual.

### Plasmids/cloning.

DNA sequences encoding the full-length human FLAG-PDE6C, mouse AIPL1-HA, EGFP-Pγ, and AIPL1-HA-IRES-EGFP-P (the AIPL1-P vector) were cloned into a mammalian expression vector, pcDNA3.1(+), as previously reported (20, 35). The R565Q mutation was introduced into the PDE6C vector using the QuikChange (Agilent) site-directed mutagenesis protocol provided by the manufacturer. The sequences of all constructs were verified by automated DNA sequencing at the University of Iowa DNA Core Facility.

### Cell culture.

HEK293T cells (293T, ATCC, CRL-3216) were cultured and maintained in DMEM containing 10% FBS (Thermo Fisher Scientific). Cells were cotransfected with pcDNA3.1(+) plasmids harboring either PDE6C and AIPL1 (1 μg each), or PDE6C and EGFP-Pγ (1 μg each), or PDE6C and the AIPL1-Pγ vector (1 μg each), using FuGene6 (Promega) according to the manufacturer’s instructions. Cells were collected 48 hours after transfection. Cell lysates, prepared in 20 mM Tris-HCl buffer (pH 7.5) containing 120 mM KCl and 1 mM MgCl_2_ and 1× EDTA-free protease inhibitor cocktail (Roche), were analyzed by immunoblotting for protein expression and assayed for PDE activity. For membrane fractionation, lysates of HEK293T cells cotransfected with PDE6C (or PDE6C^R565Q^), AIPL1, and Pγ were prepared in isotonic buffer (20 mM Tris-HCl buffer, pH 7.5, containing 120 mM KCl and 1 mM MgCl_2_ and 1× EDTA-free protease inhibitor cocktail) and centrifuged at 125,000*g* for 30 minutes at 4°C in a Beckman Optima TLX Ultracentrifuge. The pellet thus obtained was further resuspended in hypotonic buffer (5 mM Tris-HCl, pH 7.5, 1 mM MgCl_2_, and 1× EDTA-free protease inhibitor cocktail) and centrifuged at 125,000*g* for 30 minutes at 4°C to obtain hypotonic supernatant. The extracted pellet was resuspended in the same hypotonic buffer and homogenized in a 1.5-ml tube using a disposable pestle. For immunofluorescence, 48 hours after transfection cells were seeded onto poly-D-lysine–coated (MilliporeSigma) (0.1 mg/ml) 4-well chambered glass slides and allowed to grow for an additional 24 hours before fixation with 4% formaldehyde.

### Immunoblotting.

Proteins separated by 4%–12% SDS-PAGE were transferred to a nitrocellulose membrane using the iBlot Western blot kit (Invitrogen) and analyzed using mouse monoclonal anti-FLAG (MilliporeSigma, F3165-1MG, 1:2000 dilution), mouse anti-HA (BioLegend, clone 16B12, catalog 901502, 1:1000 dilution), and mouse anti-GFP (Santa Cruz, SC-9996, 1:2000 dilution) primary antibodies. The antibody-antigen complexes were detected using horseradish peroxidase–conjugated bovine anti-mouse (Santa Cruz, SC-2371, 1:10,000 dilution) secondary antibody and enhanced chemiluminescence (ECL) reagents obtained from GE Healthcare. PDE6C and PDE6C^R565Q^ protein expression levels were quantified using analysis of immunoblot data with NIH ImageJ software according to the user guide.

### Immunofluorescence.

Transfected HEK293T cells on chambered glass slides were prepared for immunofluorescence as previously reported (20, 35). Briefly, fixed and permeabilized cells were blocked for 60 minutes in blocking solution (1% BSA in 1× PBS) and then incubated at 4°C overnight with either rabbit anti-PDE6C (71) or mouse anti-HA antibodies diluted in the blocking solution. After washes with PBS, cells were incubated in the dark for 1 hour in either Alexa Fluor 568–conjugated goat anti-rabbit (Life Technologies, A-11011) or Alexa Fluor 488–conjugated goat anti-mouse (Life Technologies, A-11017), or Alexa Fluor 647–conjugated F(ab’)2-goat anti-mouse IgG (H+L) secondary antibody (Thermo Fisher Scientific, A-21237) diluted in the blocking solution. After washes with PBS, where indicated, the nuclei were counterstained with To-Pro-3 (Thermo Fisher Scientific) for 30 minutes in the dark at 25°C. Cells were mounted using Vectashield mounting medium (Vector Laboratories, Inc.) and imaged using Plan-Neofluar ×40/1.3 oil lens and a LSM 510 confocal microscope (Zeiss).

### PDE6 activity assay.

The cGMP hydrolysis was measured in cell extracts obtained from HEK293T cells 48 hours after transfection. Where indicated, samples were treated with 0.1 mg/ml TPCK-Trypsin (MilliporeSigma) on ice for 10 minutes to selectively degrade Pγ, after which trypsin was inhibited with the addition of 10-fold excess of soybean trypsin inhibitor (MilliporeSigma) and incubated for 5 minutes at 25°C. Cell extracts (protein concentration 3–6 mg/ml) were diluted 4–400 fold into 40 μl (final volume) of 20 mM Tris–HCl (pH 7.5) buffer containing 120 mM NaCl, 2 mM MgSO_4_, 1 mM 2-mercaptoethanol, 0.1 U bacterial alkaline phosphatase, and 10 μM [^3^H]cGMP (100,000 cpm) (PerkinElmer) for 15 minutes at 37°C. The reaction was stopped by the addition of AG1-X2 cation exchange resin (0.5 ml of 20% bed volume suspension). Samples were incubated for 6 minutes at 25°C with occasional mixing, and spun at 10,000*g* for 3 minutes. A quantity of 0.25 ml supernatant was removed for counting in a scintillation counter.

### Statistics.

Quantitative comparisons of ERG measurements (Figure 2) and foveal thickness (Figure 4) between groups were made using the 2 tailed Student’s *t* test. Optical coherence tomography semiautomated segmentation was compared quantitatively using the Wilcoxon test. Enzymatic assays were analyzed by 1-way ANOVA with Tukey’s multiple comparisons follow-up test. In all cases, *P* values less than 0.05 were deemed significant.

### Study approval.

The present study with animals was reviewed and approved (protocol 20145) by the Institutional Animal Care and Use Committee of the University of California at Davis.

## Author contributions

AM and SMT conceived and designed the project, and acquired, analyzed, and interpreted the data. AM drafted the manuscript with substantial participation from SMT. J. Roberts, RC, NOA, CJM, JTS, and J. Rogers participated in study conception; acquisition, analysis, and interpretation of data; and critical revision and editing of the manuscript. SK, YH, GY, RAH, YL, MR, SD, QL, OP, JW, LG, AC, KNG, and KB participated in data acquisition and revision of the manuscript.

## Supplementary Material

Supplemental data

Supplemental Table 1

Supplemental Video 1

## Figures and Tables

**Table 1:**
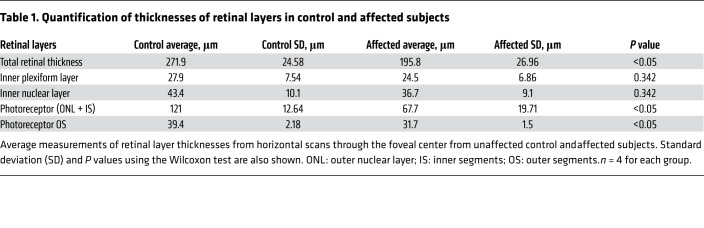
Quantification of thicknesses of retinal layers in control and affected subjects

## Version 1 01/22/2019

Electronic publication

## Version 2 02/01/2019

Print issue publication
