# Supplementary material for: A nonhuman primate model of inherited retinal disease
Source: J Clin Invest. 2019 Jan 22;129(2):863–74. doi: 10.1172/JCI123980 (PMC6355306; doi:10.1172/JCI123980)
Supplement: Supplemental data [file jci-129-123980-s291.pdf]

Supplemental Data

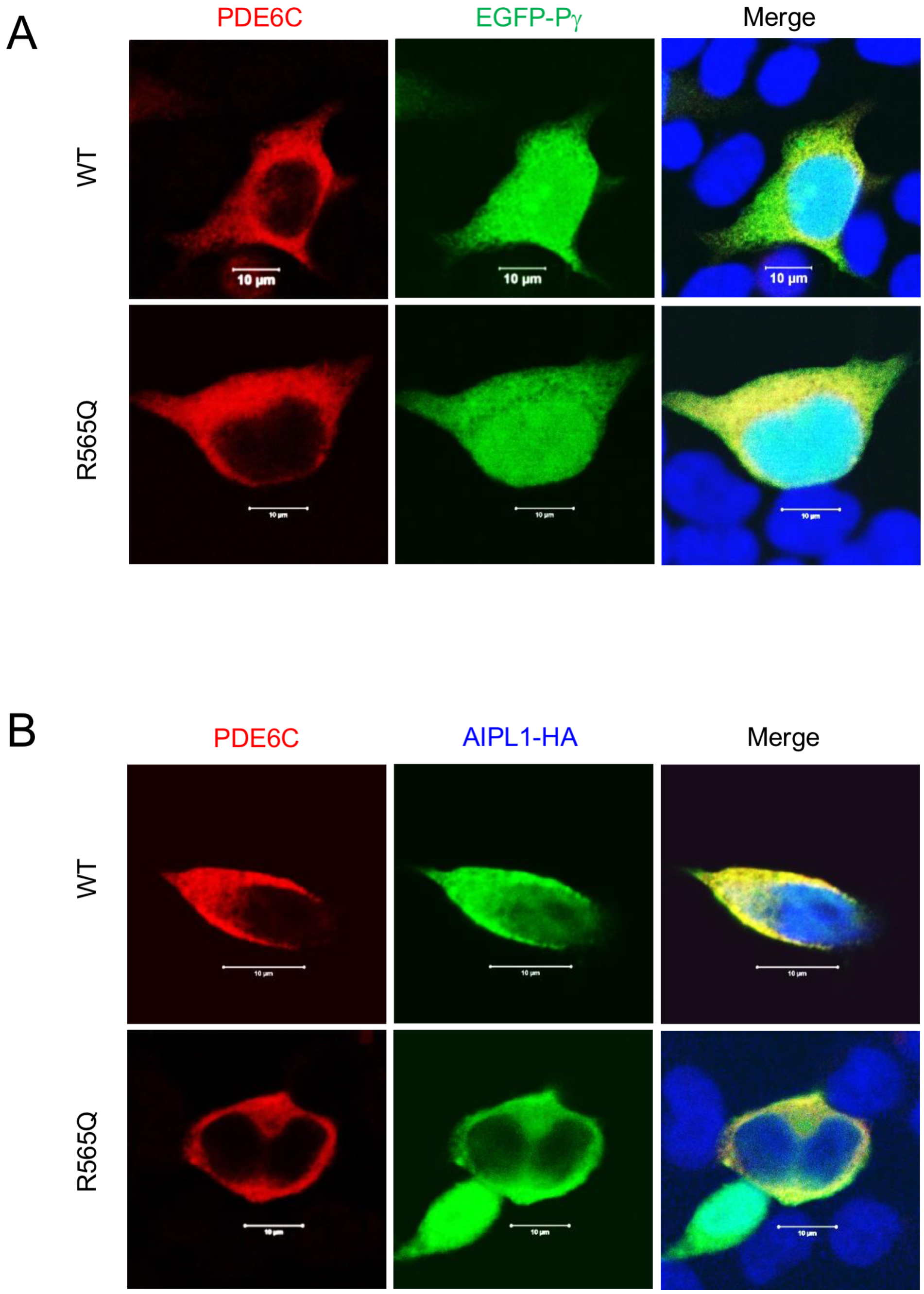

Supplemental Figure 1. Subcellular distribution of PDE6C and its R565Q mutant co-expressed with Pγ or AIPL1 in HEK293T cells.
Confocal immunofluorescence images of HEK293T cells (A) co-transfected with PDE6C or R565Q (red, anti-PDE6C) and EGFP-Pγ (green, EGFP fluorescence). (B), co-transfected with PDE6C (red, anti-PDE6C) and AIPL1 vector (AIPL1: green, anti-HA) Blue, TO-PRO3 nuclear stain. The findings suggest that the distributions of PDE6C and R565Q in HEK293T cells are qualitatively similar.

```
|PDE5AB_Zebrafish       LEVLSYHASAAEEETRALQVTAAATIPSAQSLRLMDYSFSDFDLTDAETTQATIRMFVDL   580
|PDE5_CHICK             LEVLSYHASAAEEESRELQVTAAAVVPSAQSLNLTDFNFSDFELSDFETTLCTIRMFTDL   574
|PDE5A_MOUSE            LEVLSYHASAAEEETRELQALSAAVVPSAQTLKITDFSFSDFELSDLETALCTIRMFTDL   572
|PDE5A_HUMAN            LEVLSYHASAAEEETRELQSLAAAVVPSAQTLKITDFSFSDFELSDLETALCTIRMFTDL   582
|PDE5A_Green_Monkey     LEVLSYHASAAEEETRELQSLAAAVVPSAQTLKITDFSFSDFELSDLETALCTIRMFTDL   582
                        **************:* **  :**.:****:*.: *:.****:*:* **: .*****.**

|PDE5AB_Zebrafish       KLVQNFQIKYKVPSLCQWILSVKKNYRKNVVYHNWRHAFNTSQCMFAVLKSGRVQNNLSD   640
|PDE5_CHICK             NLVQNFQMKH--EVLCRWILSVKKNYRKNVAYHNWRHAFNTAQCMFAALKSGKIQSKLTD   632
|PDE5A_MOUSE            NLVQNFQMKH--EVLCRWILSVKKNYRKNVAYHNWRHAFNTAQCMFAALKAGKIQNKLTD   630
|PDE5A_HUMAN            NLVQNFQMKH--EVLCRWILSVKKNYRKNVAYHNWRHAFNTAQCMFAALKAGKIQNKLTD   640
|PDE5A_Green_Monkey     NLVQNFQMKH--EVLCRWILSVKKNYRKNVAYHNWRHAFNTAQCMFAALKAGKIQNKLTD   640
                        :******:*:    **:*************.**********:*****.**:*::*.:*:*
```

Supplemental Figure 2. Amino acid sequence alignment shows the conservation of Aspartate (D) and Arginine (R) in the catalytic domain of PDE5 across several vertebrate species.

Supplemental File 1. The in silico analysis of the effect of the corresponding Human variant LRP5,c.G1694A;p.R565Q. Please see the table header for the meaning of each column. (downloadable Excel file)
